# The Assessment of Cholinesterase from the Liver of *Puntius Javanicus* as Detection of Metal Ions

**DOI:** 10.1155/2014/571094

**Published:** 2014-10-27

**Authors:** Mohd Khalizan Sabullah, Mohd Rosni Sulaiman, Mohd Yunus Abd Shukor, Mohd Arif Syed, Nor Aripin Shamaan, Ariff Khalid, Siti Aqlima Ahmad

**Affiliations:** ^1^Department of Biochemistry, Faculty of Biotechnology and Biomolecular Sciences, Universiti Putra Malaysia (UPM), 43400 Serdang, Selangor, Malaysia; ^2^Faculty of Food Science and Nutrition, Universiti Malaysia Sabah, Jalan UMS, 88400 Kota Kinabalu, Sabah, Malaysia; ^3^Faculty of Medicine and Health Sciences, Universiti Sains Islam Malaysia, 13th Floor, Menara B, Persiaran MPAJ, Jalan Pandan Utama, Pandan Indah, 55100 Kuala Lumpur, Malaysia; ^4^Biomedical Science Program, Faculty of Biomedicine and Health, Asia Metropolitan University, 43200 Cheras, Selangor, Malaysia

## Abstract

Crude extract of ChE from the liver of* Puntius javanicus* was purified using procainamide-sepharyl 6B. S-Butyrylthiocholine iodide (BTC) was selected as the specific synthetic substrate for this assay with the highest maximal velocity and lowest biomolecular constant at 53.49 *µ*mole/min/mg and 0.23 mM, respectively, with catalytic efficiency ratio of 0.23. The optimum parameter was obtained at pH 7.5 and optimal temperature in the range of 25 to 30°C. The effect of different storage condition was assessed where ChE activity was significantly decreased after 9 days of storage at room temperature. However, ChE activity showed no significant difference when stored at 4.0, 0, and −25°C for 15 days. Screening of heavy metals shows that chromium, copper, and mercury strongly inhibited* P. javanicus* ChE by lowering the activity below 50%, while several pairwise combination of metal ions exhibited synergistic inhibiting effects on the enzyme which is greater than single exposure especially chromium, copper, and mercury. The results showed that* P. javanicus* ChE has the potential to be used as a biosensor for the detection of metal ions.

## 1. Introduction

In biological systems, heavy metals are present in the form of metal ions and only in trace amounts. Some heavy metals are required for biochemical reactions but [1–3] high concentrations of heavy metals in the body may adversely affect the physiological function due to bioaccumulation of heavy metals at vital organs and overproduction of reactive oxygen species [4–6]. Glusker at al. mentioned that the normal function of metal ions is to facilitate the formation of enzyme-substrate complex, but metal ions tend to bind at active sites or allosteric sites and react with terminal −OH and –SH functional groups, eventually leading to conformational alterations and failure of substrates to bind at the specific site of the enzyme [7]. Metal ions such as copper, cadmium, mercury, and chromium are also considered as neurotoxic compounds that are capable of inhibiting the activity of enzymes such as ChE [8–11].

ChE, which is abundant in brain tissue, plays a role in signal termination at cholinergic synapses by rapid hydrolysis of the neurotransmitter acetylcholine. This enzyme is also present in the liver to act in detoxification [12–16]. The inhibition of cholinesterase with nerve agents, especially heavy metals, causes the accumulation of acetylcholine at the synaptic cleft and interrupting the nervous transmission, eventually leading to paralysis and death [17].

Copper and mercury are known to cause abnormalities in fish such as less feed intake and decreasing swimming activity [18–22]. Previous studies used fish as a biomarker tool through application of ChE enzyme for detection of heavy metal exposure [8, 9, 11, 23]. Emerging method of inhibitive enzyme-based assay of heavy metal was facilitated to obtain the data with low cost, low time consuming, and simple skill technique [24]. Thus, in this study, cholinesterase was isolated from the liver of local freshwater fish,* P. javanicus*, in order to evaluate the inhibitory effect of metal ions toward the enzyme activity and ascertain its capability as a new local source of biomarker of those toxicants.

## 2. Materials and Methods

### 2.1. Chemicals

Silver (ii), arsenic (v), cadmium (ii), chromium (vi), copper (ii), mercury (ii), plumbum (ii), zinc (ii), acetylthiocholine iodide (ATC), 5,5′-dithiobis (2-nitrobenzoic acid) (DTNB), BTC, and propionylthiocholine iodide were purchased from Sigma-Aldrich.

### 2.2. Extraction and Purification (Affinity Chromatography)

Fish weighing 300–500 g and approximately 20 cm in length were obtained from Aquaculture Development Center, Bukit Tinggi, Pahang.* P. javanicus* liver was dissected and weighed. The liver was homogenized using mortar and pestle in 0.1 M sodium phosphate buffer, pH 8.75 containing 1 mM phenylmethylsulfonyl fluoride (the liver and the buffer ratio was 1 : 4) using an Ultra-Turrax T25 homogeniser. The extract was centrifuged at 100,000 ×g in a Sorvall Ultra Pro 80-TH-641 for one hour at 4°C. The supernatant was collected and stored at −25°C and left to thaw at the ambient temperature for the purification process. 400 *μ*L of the supernatant was loaded into the affinity column containing procainamide-sepharyl 6B with the dimensions of 16 mm diameter and 50 mm height. Washing stage was carried out by loading 5 mL of washing buffer; 20 mM sodium phosphate buffer pH 7.0 onto the column with the flow rate calibrated at 0.2 mL/min. This stage is important to eliminate the unbounded protein to the matrix from the column. For eluting buffer, 20 mM sodium phosphate buffer pH 7.0 containing 1 M NaCl was then loaded to elute the ChE of* P. javanicus* which is bounded to the affinity matrix. 1 mL fractions was collected and assayed for enzyme activity and protein concentration determination. The fraction that exhibited the highest activity was subsequently concentrated and dialysed using Sartorius Vivaspin 20 at 2500 rpm at 4°C for 10 minutes. Purified ChE was stored at −25°C.

### 2.3. ChE Activity and Protein Content Determination

The activity of* P. javanicus* ChE was measured using the method of Ellman et al. with slight modifications, using 96-well microplate at the wavelength of 405 nm [25]. 200 *μ*L of sodium phosphate buffer (0.1 M, pH 7.0), 20 *μ*L of DTNB (0.1 mM), and 10 *μ*L ChE were loaded into the microplate wells and incubated for 15 minutes. Then, 20 *μ*L of ATC (2.5 mM) was added to the mixture and incubated for 10 minutes. ChE activity was expressed as the amount of substrate (*μ*M) broken down by ChE per minute (U) with the extinction coefficient of 13.6 mM^−1^ cm^−1^ while the specific activity is given as *μ*mole/min/mg of protein or U mg^−1^ of protein. Protein content determination was measured using the method developed by Bradford [26]. Bovine serum albumin (BSA) was used as a standard for the quantitative determination of the protein. All of the tests were carried out in triplicates and the assays were run in the dark.

### 2.4. Optimal Assay Determination

#### 2.4.1. Substrate Specificity

The substrate specificity for* P. javanicus* ChE was determined in sodium phosphate buffer (0.1 M, pH 7.0), at the ambient temperature with different synthetic substrates, namely ATC, BTC, and PTC, at concentrations ranging from 0.1 to 2.5 mM. The reading at 405 nm was recorded 10 minutes after the substrate was added into the assay reaction mixture. Michaelis-Menten curves were plotted to determine the maximal velocity (*V*_max⁡_) of ChE activity and biomolecular constant (*K*_*m*_) using GraphPad Prism Software version 5.

#### 2.4.2. pH and Temperature Profile and Storage Condition

ChE was incubated in different buffers using an overlapping buffer system consisting of 0.1 M acetate buffer (pH 3 to 5.5), 0.1 sodium phosphate buffer (pH 5.5 to 8), and 0.1 M tris-HCl buffer (pH 7 to 10). The optimal temperature of the assay was determined by incubating the reaction mixture in different temperatures ranging from 15 to 50°C. Beyond this temperature, the ChE was considered to be fully denatured. Optimal storage condition was tested by storing the purified ChE at room temperature 4.0, 0, and −25°C. Absorbances were recorded for every three days until 15 days of storage.

### 2.5. The Effect of Metal Ion (Single and Synergistic Effects)

The effects of eight metal ions, namely, silver (II), arsenic (V), cadmium (II), chromium (VI), copper (II), mercury (II), plumbum (II), and zinc (II) ChE of* P. javanicus* were tested. These metals were selected due to their capability to cause a negative impact to the environment. The reaction mixture contained 150 *μ*L of sodium phosphate buffer (0.1 M, pH 7.5), 50 *μ*L of the metal ion with the final concentration of 5 mg/L, 20 *μ*L of DTNB (0.1 mM), and 10 *μ*L of the ChE. The mixtures were incubated for 15 minutes. 20 *μ*L of the substrate was loaded into the mixture followed by 10 minutes of incubation. The absorbance was read at the end of the incubation time at the wavelength of 405 nm. Synergistic effect studies were performed by incubating ChE under the same conditions above but using a (1 : 1) mixture of two metal ions in the assay system.

### 2.6. Statistical Analysis

The means ± standard deviations (SE) were analyzed using GraphPad Prism version 5.0. Comparison between two or more groups was calculated based on a Student's* t*-test or a one-way analysis of variance (ANOVA) with post hoc analysis by Tukey's test and *P* < 0.05 was considered statistically significant [27].

## 3. Results

### 3.1. Purification

**Figure 1:**
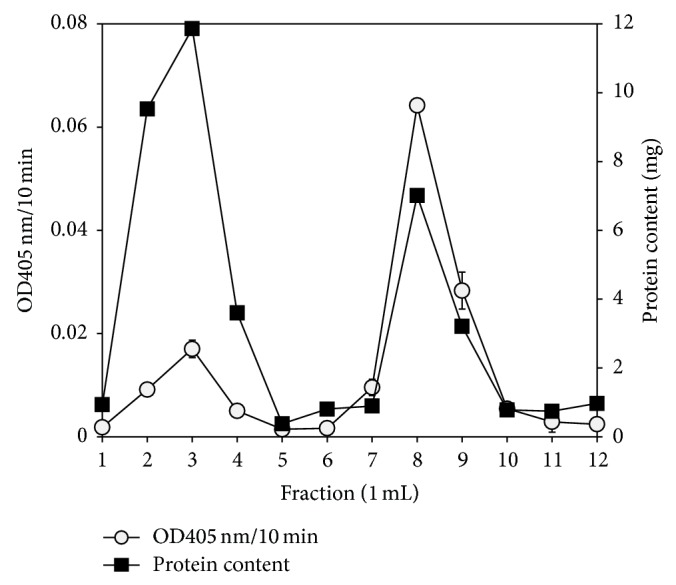
Profile of procainamide-based affinity chromatography purification on ChE from* P. javanicus* liver. Error bars represent mean ± standard error (*n* = 3).

Figure 1 shows the purification data of* P. javanicus* ChE from the procainamide-based affinity chromatography. A large amount of protein and a low ChE activity were detected at the washing stage (fractions 1 to 6) and this finding was due to column overloading. ChE was isolated at the eluting stage of the purification with high ionic strength 20 mM of sodium phosphate buffer pH 7.5, containing 1.0 M NaCl. A fraction with the highest ChE activity was collected and stored for subsequent use. Procainamide-sephacryl 6B matrix produced a ChE purification fold of 15.54 times relative to the specific activity of crude extract and recovering 38.28% of the original activity (Table 1).

### 3.2. Kinetic Study

**Figure 2:**
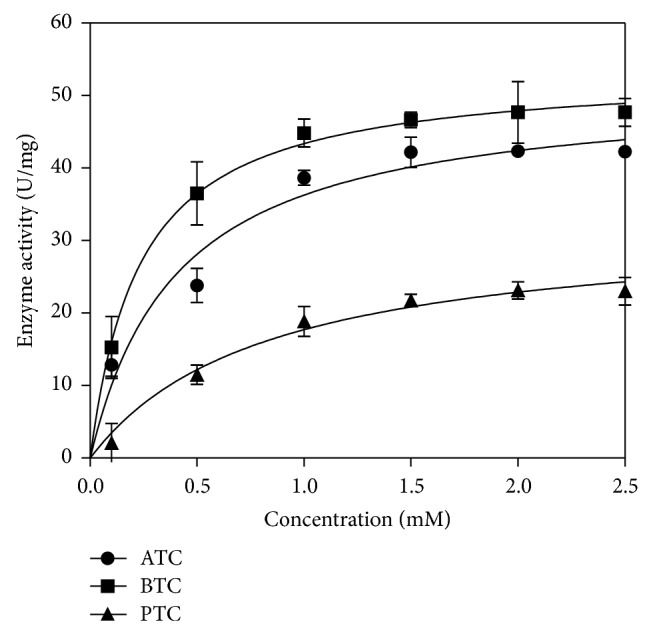
Three synthetic substrates, namely, acetylthiocholine iodide (ATC), butyrylthiocholine iodide (BTC), and propionylthiocholine iodide (PTC), with different concentrations ranging from 0 to 2.5 mM incubated with ChE to prove its specificity. Error bars represent mean ± standard error (*n* = 3).

The data showed that the ChE reaction obeyed Michaelis-Menten kinetics in hydrolysing the three different substrates, namely, ATC, BTC, and PTC, at varying concentrations (Figure 2). All three of the reactions showed increasing hydrolytic activity over increasing substrate concentration. However, at above 1 mM substrate concentration the enzyme exhibited steady state. BTC recorded the lowest *K*_*m*_ values than that of ATC and PTC, indicating that the affinity of the enzyme towards BTC substrate was higher (Table 2).* P. javanicus* ChE was also recorded to hydrolyse BTC at a higher rate compared to ATC and PTC. Catalytic efficiency (*V*_max⁡_/*K*_*m*_) was calculated and BTC displayed the highest ratio and thus was selected as the preferred substrate for this assay.

### 3.3. Optimal pH and Temperature and Selection of the Storage Condition

**Figure 3:**
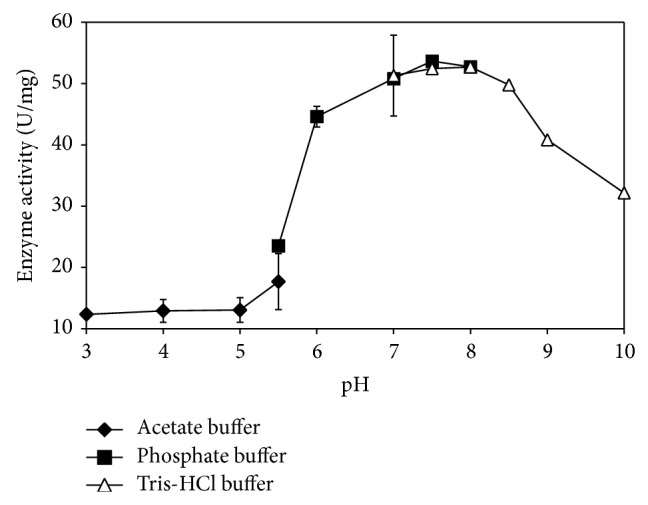
Optimisation of pH for* P. javanicus* ChE. Error bars represent mean ± standard error (*n* = 3).

**Figure 4:**
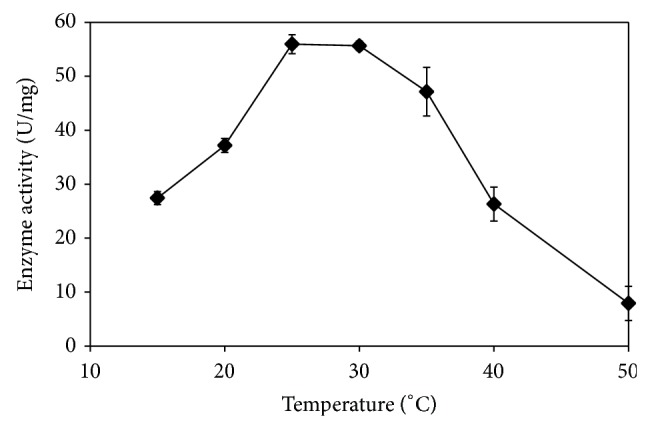
Optimisation of temperature for ChE from* P. javanicus*. Error bars represent mean ± standard error (*n* = 3).

**Figure 5:**
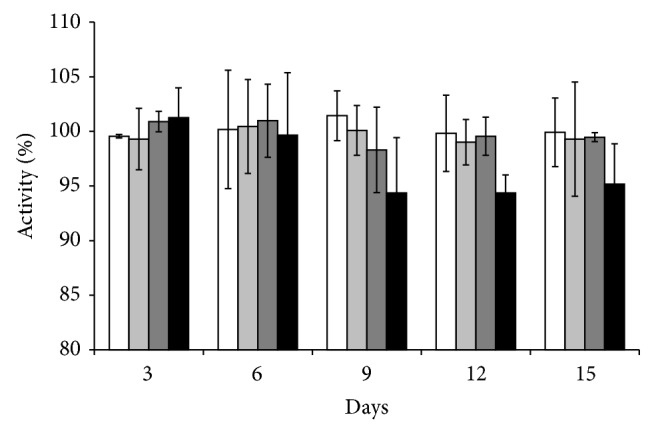
Storage condition of ChE at different temperature: (black) room temperature, (dark grey) 4°C, (light grey) 0°C, and (white) −25°C. Error bars represent mean ± standard error (*n* = 3).

The purification of ChE was carried out to determine the optimal pH and to identify the effects of extremely high and extremely low pH towards ChE activity.  Figure 3 shows the optimal pH for* P. javanicus* ChE to be between pH 7.0 and 8.0 of sodium phosphate and Tris-HCl buffer. For 0.1 M sodium phosphate buffer, pH 7.5 was selected as the highest mean point of the data compared to other buffers although analysis of each group of triplicates shows no significant difference of ChE activity (*P* > 0.05). The study on the effect of temperature (Figure 4) showed that the optimum ChE activity was in the range of 25–35°C and both of the mean points displayed no significant difference (*P* > 0.05). This bell shaped curve shows that at low temperatures, the ChE activity was retarded but rose as the temperature was increased until reaching the given velocity. Then, the activity decreased sharply at higher temperatures. The optimum temperature for* P. javanicus* ChE coincides with the ambient temperature usually encountered in Malaysia. ChE that were separately stored at room temperature, 4.0, 0, and −25°C, showed no decrease or increase of the enzyme activity (*P* > 0.05) during the first 6 days. However, after 9 days, the ChE activity was significantly reduced (*P* < 0.05) by 6% while no reduction was seen in other storage conditions after 15 days of storage (Figure 5).

### 3.4. Metal Ion Inhibition Study

**Figure 6:**
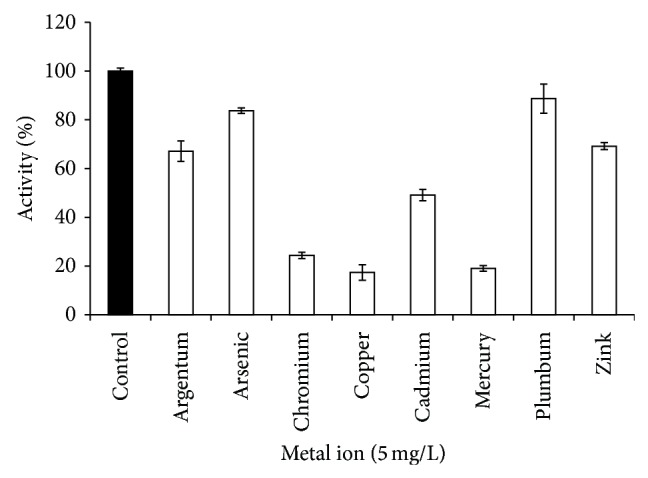
Percentage of enzyme activity after inhibition by heavy metals at 5 mg/L final concentration. Error bars represent mean ± standard error (*n* = 3).

**Figure 7:**
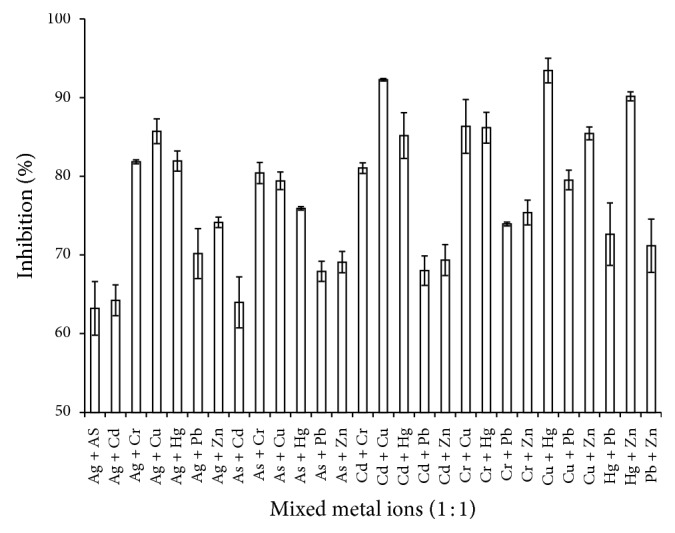
Synergistic reaction of* P. javanicus* ChE with the pairwise metal ion. Error bars represent mean ± standard error (*n* = 3).

All of the optimal assay parameters were combined and* P. javanicus* ChE was tested by incubating it with the selected metal ions with the concentration of 10 mg/L.  Figure 6 shows that ChE was inhibited* in vitro* by silver (Ag^2+^), arsenic (As^5+^), chromium (Cr^6+^), copper (Cu^2+^), cadmium (Cd^2+^), mercury (Hg^2+^), lead (Pb^2+^), and zinc (Zn^2+^) by lowering the activity to 67.15, 83.76, 24.38, 17.37, 49.144, 19.07, 88.68, and 69.20%, respectively. Copper, chromium, and mercury displayed the highest inhibition, lowering the activity of ChE to less than 50% but showing no significant difference (*P* > 0.05) compared to each other. Previous studies also report that copper and mercury are strong ChE inhibitors [28, 29]. Although the data showed that arsenic and lead caused lower inhibition of ChE activity, other studies have proved the toxicity of these metals towards ChE [30–32]. Thus, at the concentration of 5 mg/L, it can be summarised that the toxicity of the tested metals is as follows: Cu ≤ Hg ≤ Cr < Cd < Ag ≤ Zn < As ≤ Pb. Synergistic inhibitory effects were observed when the tested heavy metals were paired together (Figure 7) such for Cd + Cu, Cu + Hg and Hg + Zn where the inhibitions were more than 90%. Slight significant (*P* < 0.05) increases were recorded for Cr + Cu, Cr + Hg, Ag + Cu, Cu + Zn, Cd + Hg, Ag + Cr, Cd + Cr, As + Cr, and As + Cr, while statistical analysis on Ag + Hg, Cu + Pb, As + Cu, As + Hg, Cr + Zn, and Cr + Pb showed no significant effects compared to single exposure of Cr, Cu, and Hg. However, these tested metal ion mixtures were able to inhibit ChE activity more than 50%.

## 4. Discussion

### 4.1. Enzyme Parameter

The presence of ChE in fish liver is well known [33, 34]. ChE is also present in other organs such as kidney [34], muscle [35], plasma, and brain tissues [36]. Previous studies utilised procainamide-based affinity chromatography to purify ChE in the extracted samples [37–41]. This purification is done to minimise any inference from other proteins to ensure that maximum performance can be reached by the desired purified protein [42]. Normally, high ionic strength compounds such as NaCl are needed to alter the ionic strength by lowering the binding capacity between ChE and procainamide ligand leading to desorption of ChE out from the system [43].

In this study, the isolated ChE was incubated with different substrates and concentrations. The enzyme showed typical Michaelis-Menten kinetic for the substrate concentration tested above 1 mM substrate, the enzyme showed steady state. Thus, the substrate concentration tested showed saturation of the reaction. This observation is in agreement with that of Diamant et al. 2006 [44]. Table 2 shows that BTC gave the highest *V*_max⁡_ app and the lowest *K*_*m*_ app, with the highest *V*_max⁡_/*K*_*m*_ ratio, which proved that ChE hydrolyzed BTC at the highest efficiency. Most previous studies utilised BTC from various animals such as horse, mice, and pigs as a specific substrate for liver ChE [45–47].

As shown in Figure 3, the enzyme-substrate complex formation is influenced by pH. ChE is sensitive in extremely low and extremely high pH, thus leading to the loss of its enzymatic function [48]. At low pH, high concentrations of protons disrupt the interaction of substrate toward the ChE due to the protonation of an imidazole group of histidine at the catalytic triad of the enzyme [49, 50]. Alteration of the histidine conformation may affect the ChE mechanism [51]. This situation also occurs at high pH at which the change of the substrate charge affects the binding of the enzyme and substrate. Optimum temperature is the key to stimulating the interaction of the enzyme and substrate [52]. At low temperatures, ChE activity is retarded without denaturing due to limited kinetic energy for ChE to hydrolyse BTC but the activity increases as the temperature rises to the maximum point. In this study, it was found that ChE activity achieved its maximum activity at the range of 25–30°C, but, beyond this temperature range, the activity rapidly decreased. Theoretically, high temperature causes ChE to lose its stability and function and then leads to protein denaturation [53, 54]. However, at 35 and 40°C, not enough evidence is available to prove that* P. javanicus* ChE is fully denatured unless the enzyme is reassayed after the temperature returns to normal temperature lower than the tested temperature. The report by Botté et al. [55] mentioned that the* in vivo Acanthochromis polyacanthus* ChE was significantly inhibited with the increase in temperature. But, at the recovery period, that is, when the temperature decreases to 28°C, the ChE activity slightly increases and the enzyme needs more time to get back to its normal state. Thus, further study is needed to determine whether temperature inhibition is reversible or not. The storage stability studies indicated that typical effects of temperature of storage on enzyme stability with lower temperatures increase enzyme stability. It is expected that for longer period of storage a much lower temperature such as −80°C should give better stability than higher temperatures due to the lower chances of large ice crystal formation that could denature enzyme. Storage condition is crucial to ensure the stability of ChE activity as the next test mentioned that cattle, sheep, and pig liver ChE are stable for three to six months at the storage conditions of −20 and −80°C. Horse blood ChE remained stable when stored at 5°C for 15 weeks but at 20°C the activity was significantly decreased [56]. Freezing and thawing may cause protein denaturation [57]. A study by Nigg and Knaak [58] determined a slight change of human plasma BChE after 10 cycles of freeze thawing at −70°C. However, the present study displayed no significant loss of ChE and this suggests that storage at the refrigerated conditions can maintain the stability of ChE activity for long time periods.

### 4.2. Inhibition of Metal Ion towards ChE

For* in vitro* ChE inhibition, it was determined by incubating* P. javanicus* ChE with 1 mg/L concentration of selected metal ions, namely, Ag^2+^, As^5+^, Cr^6+^, Cd^2+^, Cu^2+^, Hg^2+^, Pb^2+^, and Zn^2+^. It was found that all of the metal ions significantly inhibited ChE activity but with different percentages of inhibition. In the present study, synergistic studies were done to evaluate if the combined effects could enhance the inhibition level without changing the optimum conditions of the enzyme. Studies by Forget et al. 2002, Toman et al. 2012, and Cacciatore et al. 2012 showed that the combination of metal/pesticides, cadmium/diazinon, and azinphos-methyl oxon/chlorpyrifos oxon caused greater enzyme inhibitions compared to the individual effects [37, 59, 60]. Synergistic effect has two main concepts, concentration effect and independent action, which enhance the adverse effect of the biological system [61]. Other nerve agents such as carbamate and organophosphate inhibit ChE activity by binding through the process of carbamylation and phosphorylation at the active site and by blocking the binding of substrate [62–64]. In comparison, inhibition by metal ions is related to the binding affinity towards the amino acid side chain. Proteins containing the histidine residue is the most vulnerable to the metal binding such as by zinc and copper [65–69]. The imidazole group of histidine provides the strongest cation-*π* attraction that may interact with nitrogenous cations of substrates or free metal ions [70–73]. However, Sarkarati et al. [74] mentioned that the inhibition of ChE by metal ions is caused by the attraction of the negative charge of amino acid side chains that contain carboxyl groups such as glutamate and aspartate present at the catalytic triad of ChE, leading to structural change of the active site [75, 76]. Other amino acids such as cysteine, methionine, phenylalanine, threonine, asparagine, glutamine, tyrosine, and tryptophan also contribute in the interaction with metal cations, either at the active site or at the allosteric site of the protein [7, 77]. Copper, cadmium, and zinc have been reported to display noncompetitive inhibition behaviour towards ChE activity, while mercury has been reported to act as an irreversible inhibitor [28, 74, 78]. It can be concluded that metal ion inhibition is related to (1) blockage of the enzyme active site, (2) alteration of ChE structure, and (3) amino acid sequence variety which tend to be affected differently by the metals and other toxicants, thus preventing the formation of enzyme-substrate complex or protein denaturation, either reversible or irreversible [79, 80]. This present study has proved the capability of metal ion to inhibit the activity of* P. javanicus* ChE activity. The mechanism of heavy metals inactivation of cholinesterase by mercury has only recently been studied intensively by Frasco et al. [81]. In their work, they reported that inactivation of mercury is through its action as a sulfhydryl reacting agent. Free sulfhydryl is present at various locations of the cholinesterases from various sources with the AChE from* T. californica* being the most susceptible to mercury inactivation compared to* E. electricus* acetylcholinesterase,* D. melanogaster* acetylcholinesterase, and human butyrylcholinesterase due to the presence of free cysteine. The inhibition can be in the micromolar or the millimolar range dependent upon sources with the acetylcholinesterase from* T. californica* having a micromolar range. The mechanism of inhibition by other heavy metals tested in this work is unknown but can be speculated to act upon the catalytic triad Ser-His-Glu which is commonly conserved in both AChE and BChE [79, 80]. For instance, the imidazole side chain of the amino acid histidine plays an important role as a ligand in biological systems and can be found in a large number of metalloproteins that binds many transition metal ions including copper, nickel, and zinc [82–84]. In addition, the serine protease trypsin is strongly inactivated by the element zinc [24] suggesting that zinc probably binds to the amino acid residue serine in the catalytic triad in cholinesterases.

## 5. Conclusion

In this work, the optimum assay conditions, namely, pH and temperature for isolated* P. javanicus* ChE from procainamide-sephacryl 6B, were successfully recorded with BTC being preferred as the specific synthetic substrate throughout the study. The sensitivity of ChE inhibition by selected metal ions was determined and the results suggest possible biosensor application of the* P. Javanicus* ChE for the detection of metal ion environmental contaminants. Future work is recommended to assess the capability to detect other contaminants such detergents, dyes, pesticides, and drugs.

## Figures and Tables

**Table 1:** Purification table for purification of ChE from *P.  javanicus*. The specific activity from each step of purification is expressed in (U/mg), which means *μ*mole/min/mg of protein.

Procedure	Total protein (mg)	Total ChE activity (U)	Specific activity (U/mg)	Purification folds	Yield (%)
Crude homogenate	296.25	828.13	2.80	1	100
Supernatant (100,000 ×g; 1 h in 4°C)	77.06	688.87	8.94	3.2	83.18
Procainamide-sepharcyl 6B	6.07	263.73	43.44	15.54	38.28

**Table 2:** The comparison of the maximum velocity (*V*_max⁡_) and biomolecular constant (*K*_*m*_) for ATC, BTC, and PTC of *P.  javanicus* ChE.

	Mean point (95% confidence intervals)		
	ATC	BTC	PTC
*V*_max⁡_ (*μ*M/min/mg)	51.07 (46.19 to 55.94)	53.49 (50.12 to 56.87)	32.34 (27.01 to 37.67)
*K*_*m*_ (mM)	0.41 (0.26 to 0.56)	0.23 (0.16 to 0.31)	0.83 (0.45 to 1.20)
Catalytic efficiencies *V*_max⁡_/*K*_*m*_	0.13	0.23	0.04
